# Single-Hair Follicular Unit Transplant for Stable Vitiligo

**DOI:** 10.4103/0974-2077.79191

**Published:** 2011

**Authors:** Muthuvel Kumaresan

**Affiliations:** *Department of Dermatology, PSG Hospitals, Coimbatore, India*

**Keywords:** Follicular unit transplant, leukotrichia, stable vitiligo

## Abstract

Follicular unit transplant (FUT) is one of the surgical procedures which has been recently used to repigment a stable vitiligo patch. Single-hair FUT was done for a 30-year-old male with stable vitiligo patch on the upper lip. Repigmentation was noted in 4 weeks and complete pigmentation seen at 8 weeks. No recurrence was noted at the end of 6-month follow-up with excellent colour match. This case is presented to highlight the effectiveness of FUT in focal vitligo patch with leukotrichia.

## INTRODUCTION

Various surgical modalities are followed to repigment the vitiligo patch and follicular unit transplant (FUT) is one among them. This procedure is based on the concept of existence of undifferentiated stem cells in the hair follicle, which forms a good source of melanocytes for repigmentation. These melanocytes when grafted, then spread to surrounding depigmented epidermis. Thus, in this method, the appearance of pigmentation is delayed when compared to other modalities and the colour match is much more acceptable. This method is effective in focal vitiligo, vitiligo in non-glabrous areas and in those patches with leukotrichia.

## CASE REPORT

**Figure 1:**
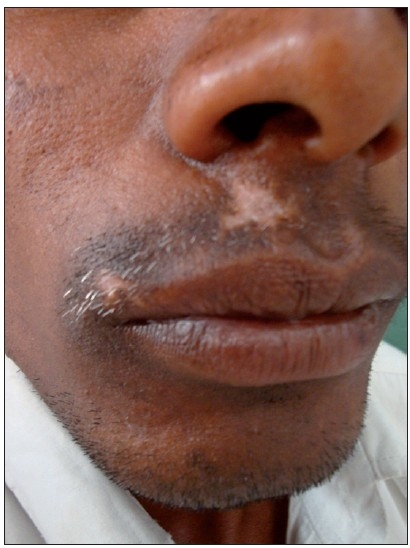
Vitiligo with leukotrichia before the surgery

**Figure 2:**
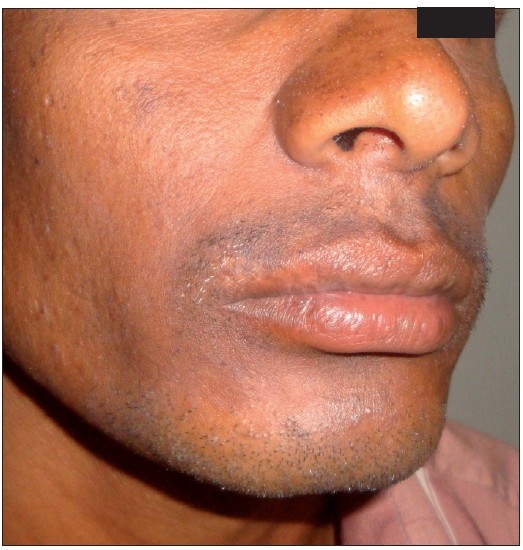
Repigmentation at the end of 8 weeks following single-hair FUT

A 30-year-old male patient presented with depigmented macules on the upper lip of 4-year duration [Figure 1]. He took various topical and oral medicines for the same without any relief. No new lesions were seen elsewhere in the body and size of the lesions remained stable for the past 2 years. On examination he had two depigmented macules of 2×2 and 1×2-cm size on the right side of upper lip in the moustache region. Leukotrichia was found to be present. A diagnosis of vitiligo was made based on the clinical findings. Since the patient did not respond to the medical management, surgical correction with FUT was suggested. Informed consent was taken. Donor hairs were harvested from the occipital scalp and then dissected into single follicular units. Donor area was closed with interrupted suturing using 3-0 nylon. The dissected follicular units were transplanted using an 18-g needle in the depigmented macules with 5-mm gap between the follicles. Paraffin gauze dressing was done for the recipient area and dressing was removed after 4 days. Patient was followed up every month. No post-operative complication was encountered. Repigmentation of the vitiligo patch was seen at the end of 4 weeks and complete pigmentation was seen at 8 weeks [Figure 2]. Colour matching was excellent. Leukotrichia at the vitiligo patch remained the same after the FUT at the end of 6 months. There was no recurrence after 6 months of follow-up.

## DISCUSSION

Various surgical procedures are practiced for treating stable vitiligo patches, e.g. punch graft, Thiersch’s graft, blister-graft, full-thickness skin graft, autologous melanocyte transplants, *in vitro* cultured epidermal grafts bearing melanocytes, micropigmentation and dermabrasion and all these techniques induce satisfactory repigmentation with some drawbacks.

FUT was introduced to repigment vitiligo patches in 1998.[1] This procedure is based on the concept of existence of undifferentiated stem cells in the hair follicle, which forms a good source of melanocytes for repigmentation. Staricco demonstrated that there were two types of pigment cells in the hair follicle, inactive and active melanocytes and the inactive melanocytes could migrate along with regenerated epidermis and would mature gradually.[2] Ortonne *et al*. postulated the existence of a melanocyte reservoir, specifically located in the lower portion of human hair follicles and they proposed that repigmentation of vitiligo was derived from the melanocyte reservoir in the hair follicles.[3] Cui *et al*.demonstrated that during the repigmentation of vitiligo the number of inactive melanocytes in the outer sheath of hair follicles significantly increased and some active melanocytes appeared in the outer root sheaths, in the hair follicle orifices and around the perifollicular epidermis.[4] Phototherapy-induced stimulation of melanocytes migration from the hair follicle reservoir is now a well-established fact. Melanocytes spread centrifugally from the infundibulum to the basal layer and recolonize the epidermis with active and functional melanocytes.[5] Regardless of the mode of treatment, repigmentation in vitiligo usually begins in the perifollicular area.

FUT for repigmentation for vitiligo has been reported earlier.[1,6,7] The diameter of pigment spread is 5-12 mm per hair grafted.[8] Pigmentation starts appearing at 4^th^ to 5^th^ week and continues upto 6 months or even longer.[8] Although the appearance of pigmentation is delayed when compared to other modalities, the colour match is much more acceptable than with other surgical modalities.[8] Even in cases of unresponsive or treatment-resistant vitiligo, grafted hairs retained the pigmentation.[1] Transformation of depigmented hairs into pigmented hairs has been reported following FUT.[1] Epilation of white hairs prior to FUT has been tried to accelerate the hair cycle so that when the new anagen hair growth started, black hair emerged quickly.[1]

The advantages of FUT in vitiligo are:[1]

1. A single hair contains more melanocytes than normally pigmented glabrous, usually gluteal area skin. Hair follicle melanocytes also seem to be more resistant to the vitiligo process.
2. Cobblestone hypertrophic scar does not appear because small bored needle is used for implantation.
3. This method is advantageous for hair restoration in a non-glabrous area.
4. Can be easily applied to a small area of vitiligo.
5. Does not produce post-operative hyperpigmentation in the grafted sites as does autologous suction blister grafts.
6. Can be performed in the eyelash area or angle of the mouth where other surgical methods, such as epidermal grafting or minigrafting, are difficult.
7. No special equipments are required for FUT.

However, this method has several limitations; hair dissection is a tedious, time-consuming and delicate procedure, needs proper training, and it is difficult to treat medium or large achromic lesions; because of the limited number of donor hairs, vitiliginous skin heals slowly with this method because the grafted melanocytes require a long time to spread.

FUT appears to be an effective method for treating localized/segmental vitiligo, especially on hairy parts of the skin, including the eyelids and eyebrows and for small areas of vitiligo. The best application of this method will be in vitiligo patches with leukotrichia. This case is presented to highlight the effectiveness of single FUT in focal vitligo patch with leukotrichia.
